# Thrombosis in Iliac Arteries

**Published:** 1891-06

**Authors:** J. C. Huilson


### THROMBOSIS OF THE ILIAC ARTERIES.

By Dr. J. Huilson.

We very seldom hear of cases of Thrombosis, and it would appear from this to be a very rare disease in actual practice. Personally, I have but little experience and that, only with a few cases in the live animal, but in the dead subject in the dissecting rooms we find it different. I think while at college, that about one out of every twenty of the horses used for dissection, exhibited thrombosis, and that too in vessels in most instances of considerable size.

One case I remember was found in the Post. Aorta near its origin, but the majority of the cases occurred in that vessel at its termination, or involving the Iliac arteries, the Museum of the college also contained many interesting specimens of this disease. It seems to me therefore from its frequency in these dead subjects that during life, the trouble must frequently remain unrecognized, and probably be the cause of lameness or disease. I will briefly consider the pathology of Thrombosis or Embolism as it might occur in any blood vessel, and then give the symptoms as exhibited especially when affecting the Post. Aorta or Iliac Arteries.

Regarding the pathology, Williams says, that a thrombosis is the coagulation of the blood in the vessels be it arteries or veins, It may proceed from inflammation of the vessels, caused by some injury, the result of this being an exudation from the walls of the vessel, forming the nucleus of the clot. The formation of the coagulum always begins at some definite fixed spot which is the source of local irritation and from this, it extends, until the artery is plugged up to its origin from the parent trunk. Portions of this clot or thrombus may often become detached from the walls or valves of the blood vessels, and be impelled onward to other parts of the circulation, these are termed embolic thrombi and may be impelled from the heart to the arteries or may form in the veins and travel to the heart, As long as the thrombus is confined to a branch vessel, there is no particular danger. The greater number of thrombi in the small branches do not content themselves with advancing up to the level of the main branch, but new masses of coagulum deposit themselves in the blood upon the end of the thrombus. Layer after layer the thrombus is prolonged beyond the mouth of the branch into the trunk in the direction of the current of the blood, and shoots out in the form of a thick cylinder, farther and farther, becoming continually larger and larger. It is these prolonged plugs that constitute the source of real danger, as the stream of blood may detach minute portions, hurry them away with it and wedge them tightly into the nearest system of arteries or capillaries. Thus many cases of sudden death that would otherwise be unexplainable are accounted for. Now regarding the symptoms. These thrombi of internal blood vessels in many cases would be impossible to recognize during life. We may have a suspicion of the trouble, but not sufficient to give a positive diagnosis. Perhaps externally, from the decreased amount of blood supplied to the part, we may detect more or less atrophy of the muscles supplied by such vessels. Thrombosis of the Iliac Arteries, the vessel supplying the circulation of the posterior extremities, however, may be recognized both by symptoms plain to the sight and by examination internally. I will endeavor to present these symptoms as shown when affecting these vessels in speaking of thrombosis. We understand of course that the blood vessel is not entirely plugged by the clot, the blood being able to trickle in most cases between the coagulum and the coat of the vessel and if both sides are affected the symptoms would be shown in both extremities.

The history of these cases will run about as follows: A horse apparently sound in every respect, when driven for a short distance, develops symptoms of lameness a weakness or slight paralysis behind, which will increase until unable to make further progress. During this time, he will break out in profuse perspiration over his body, the leg is kept in constant motion alternately raised and lowered, in a spasmodic manner. Respiration is hurried, pulse very weak and the horse seems in great pain. Another marked symptom, is on feeling the leg below the hock we find it to be of an icy coldness. If you continue to urge him, he may fall down, and after resting or lying a certain length of time he will get up and when brought to the stable stands and rest, and the symptoms disappear, however, to reappear upon the same exercise. As long as he is in the stable he shows no symptoms, but when the circulation of the blood is increased by exercise the spasm or cramps of the muscles and irritation again develops. The lameness resembles, and when first seen, might easily be mistaken for spavin, walking on the toe as in hock lameness.

The disease may sometimes, also with hasty diagnosis, be mistaken and treated for Azoturia or colic, but with such a history, a patient showing these symptoms every time he is driven or exercised, and recovering after rest, the profuse prespiration and cold extremities, the Veterinarian will be led to suspect the real trouble, and the diagnosis can be easily confirmed by an examination, per rectum. Inserting the hand into this organ searching for these large blood vessels which will be found situated beneath the last lumbar vertebra, we can feel the hard or cordy vessel and the loss of pulsation, or a fluttering sensation as of a small stream passing through a narrow space. The difference between that and the healthy artery will confirm our diagnosis.

Treatment. There is nothing I know of that will avail in this trouble. Perhaps a mild case might be found where attempts may be made to cause absorption of the clot, assisted by medicinal agents, such as iodide of potash, mercurials or alkalies, or turning out to pasture trusting to nature. But I have not yet heard of any successful treatment.

